# Multilevel relational influences on HRM practices: a cross-country comparative reflective review of HRM practices in Asia

**DOI:** 10.1057/s41291-022-00208-z

**Published:** 2022-10-21

**Authors:** Ashish Malik, Vijay Pereira, Pawan Budhwar, Fabian Jintae Froese, Dana Minbaeva, James Sun, Anh Tuan Nguyen, Shanzi Xue

**Affiliations:** 1 University of Newcastle, Callaghan, Australia; 2 NEOMA Business School, Mont-Saint-Aignan, France; 3 Aston University, Birmingham, UK; 4 University of Goettingen, Platz der Goettinger Sieben 5, 37073 Goettingen, Germany; 5 King’s College London, London, UK; 6 University of Auckland, Auckland, New Zealand; 7 Vietnam National University, Hanoi, Vietnam

**Keywords:** HRM practices, Cross-country comparative review, Asia, Convergence, Divergence

## Abstract

In this paper, we respond to the calls for context-specific scholarship and research on human resource management (HRM) in Asia. We provide an overview of and key insights into HRM in five Asian countries, representing five key regions: China (East Asia), India (South Asia), Kazakhstan (Central Asia), United Arab Emirates (West Asia), and Vietnam (Southeast Asia). Based on our comprehensive, pan-Asian review, we develop a reflective, comparative, and relational understanding of HRM practices. In doing so, we group the myriad contextual influences on the shaping of HRM practices at three broad levels: macro-, meso-, and microlevel influences. Specifically, we propose that influences from regionalization of economies, national business systems, industry, multinational enterprises, and individual-level predispositions collectively shape and variously influence the nature and extent of HRM practices. By considering the findings of prior research on convergence and divergence, we offer a nuanced perspective wherein each country and region in Asia possesses a distinct amalgam of national business systems, and where HRM practices respond to multilevel influences in varied ways.

## Introduction

Recently, the contribution of the Asian region to global economic activity has continued to grow when other developed markets’ trade outputs have shrunk (Asian Development Bank, 2015). This trend reflects the increasing number of emerging markets from the Asian region (Chattopadhyay et al., 2012; Mishra & Sohani, 2020; The Economist, 2020), along with its trend of attracting increasing levels of foreign direct investment (FDI) (UNCTAD, 2020). Although the global economy has been negatively influenced by the COVID-19 crisis and the Russian invasion of Ukraine in the past two years (Liu & Froese, 2020; Markus, 2022), Asian economies are expected to continue to play an important role in the long-term (OECD, 2022).

Given the importance of Asian economies, substantial research has investigated Asian businesses and their management (Froese et al., 2022). Increasingly, wisdom prevails in support of “context-specific research” as Western management approaches either fail to explain, or only partially explain, business and management practices in the Asian context (Froese et al., 2019; kuk Kang & Fornes, 2017; Schuler et al., 2002). As such, there are calls for context-specific scholarship and research, especially within the domain of HRM-related research (see, e.g., Budhwar & Sparrow, 2002; Morishima, 1995; Schuler et al., 2002; Sekiguchi et al., 2016), as well as explorations that focus on indigenous management constructs suited to specific geographic settings (Cappelli et al., 2010; Chung et al., 2014; Froese et al., 2020). Some studies have even portrayed differences within a single country (Pereira et al., 2017) or the same sector of a country (Malik et al., 2021a, 2021b). Large countries, such as China (Sheldon et al., 2014a, 2014b; Zhu & Warner, 2019) and India (Mishra & Sohani, 2020; Panda et al., 2022; Pereira & Malik, 2013, 2015), can have substantial heterogeneity within their borders. 

We propose that the nature and extent of such scholarship about HRM in the Asian context are still in an early stage (e.g., Cooke et al., 2020; De Cieri et al., 2022; Do et al., 2020; Zhu et al., 2007a, 2007b). Our focus in this paper is on representative countries of the Asian continent that have witnessed a boom in FDI since they liberalized their economies (though of varying levels). Attendant to FDI is the arrival of foreign firms and their global HRM practices, some of which are eventually adopted by host-country firms (Beresford, 2008; Budhwar & Varma, 2011a, 2011b; Farndale et al., 2022; Vo, 2009). Unfortunately, the vast majority of HRM research tends to adopt a largely objective view of the world when it comes to leading and managing the people in organizations (Jackson et al., 2014; Kaufman, 2015; Meyer, 2014), almost to the point where prescriptive formulations must precede any descriptive analysis or evaluation. The importance of context-specific HRM research has been highlighted in several studies (Bader et al., 2021; Budhwar et al., 2016), isolating multiple levels of contextual influences on the shaping of the nature and extent of HRM practices (Brewster et al., 2016; Cooke, 2017; Morley et al., 2021; Pereira et al., 2017; Rowley & Benson, 2002).

When considering the myriad contextual influences affecting the shaping of HRM practices, we group these at three broad levels: Macro-, meso-, and microlevel influences. Specifically, we assert that influences from the regionalization of economies, national business systems (incorporating divergent cultural and institutional influences and converging national business practices with managerial ideologies), industry, global multinational enterprises (MNEs), and finally, individual-level predispositions collectively shape and variously influence the nature and extent of HRM practices. A view that these multiple levels of influence prevail has been widely acknowledged, for example, in studies on the influence of regionalization of economies (Ferner & Quintanilla, 1998; Li & Hendrischke, 2020), national business systems (Do et al., 2020; Whitley, 1999; Witt & Redding, 2014), industry (Malik et al., 2017, 2020; Milliman et al., 1991; Zhang et al., 2022), global MNEs (e.g., Ferner, 1997; Rosenzweig & Nohria, 1994), and individual-level predispositions (Froese, 2013; Hitotsuyanagi-Hansel et al., 2016; Minbaeva et al., 2012; Singh et al., 2021) on HRM practices. Nevertheless, most research is generally undertaken at one or at most two levels of analysis (Li et al., 2022). We present in this paper a reflective, comparative, and relational understanding of HRM practices in five key Asian regions: East, Central, West, South, and Southeast. More specifically, we focus on the following countries (these are *representative* of the above macrolevel regions, and as authors, we represent these countries as former and/or current nationals/residents who have been actively researching HRM in these countries over the years): China, India, Vietnam, Kazakhstan, and the United Arab Emirates (UAE). While Asian countries, to some scholars, might offer opportunities for greater convergence than divergence, we take a nuanced view wherein each country and region of Asia offers a distinct amalgam of national business systems and ways in which HRM practices respond to multilevel influences.

In keeping with the proposals of several leading scholars (Budhwar & Sparrow, 2002; Budhwar & Varma, 2014; De Cieri et al., 2005; Schuler et al., 2002; Zhang, 2012), we assert that any prescriptive understanding, especially for applied fields of study and practice, such as HRM, must proceed with thick descriptions of the context. What do managers in Asian organizations do in relation to their HRM practices? How do they manage the multiple levels of influences such as those outlined above in shaping the nature and extent of their HRM practices? Is there convergence for some and divergence for other practices? If so, why? If not, why? We develop such descriptive understanding through our review of extant literature on the above theme, as well as by applying our subjective experience as scholars with decades of work and research in the field of HRM. We consider that the latter set of perspectives is equally relevant and invaluable as it can provide critical insights into why a phenomenon is unfolding in a certain way. Only when we have an adequate descriptive understanding of each region can we begin to offer further thematic and relational insights into each country in terms of how the multiple levels of influence shape and affect indigenous HRM practices to converge, diverge, or produce a novel amalgam of HRM practices. To that end, in this paper, we address the following research questions: 

### RQ1

How do global, regional, and national business systems, industry, and individual influences shape the nature and extent of HRM practices in five Asian regions?

### RQ2

Based on the above influences, what are the outcomes in terms of indigenous and global HRM best practices?

### RQ3

Does the above analysis suggest increasing evidence of convergence, divergence, crossvergence, or multivergence in HRM practices?

The rest of the paper is organized as follows. To answer the study’s three research questions, we begin by introducing our methodology and stating the guiding theoretical lenses through which we analyze the nature and extent of HRM in Asia. Next, we offer a description of the indigenous HRM practices in each country and analyze the relational multilevel influences shaping these indigenous HRM practices. Next, we provide a discussion of the outcomes and analyze evidence that suggests convergence, divergence, crossvergence, or multivergence (Froese, 2013; Froese et al., 2020; Malik et al., 2021a) in HRM practices. Based on the above, we close with a discussion of where future research and scholarship are needed.

## Methodology

This article is reflective in nature as it is not only *representative* of the macrolevel regions of Asia but also authored by scholars who represent these countries as former and/or current nationals/residents who have been actively researching HRM in these countries over the years. Since the HRM literature on Asia is widely dispersed, we set out to conduct a structured review (Tranfield et al., 2003). Based on the approach taken to produce a recent review by Budhwar et al. (2018) on HRM in the Middle East, we grounded our search and selection of published literature on three critical parameters: R*elevance, quality,* and *recentness*. In terms of the *relevance* parameter, we explored the extant and relevant literature to obtain an overview of the number of articles published, for which Asian region/country (East, Central, West, South, or Southeast), and concerning which thematic areas. More specifically, we used ProQuest to search for articles and studies related to the key HRM themes and terms relevant to our analysis (based on the three research questions we framed above). The preconditions for this analysis were that each paper was peer-reviewed and available in the full-text format. In practice, we ran a number of Boolean filter searches using relevant terms for the topic of interest and thematic area, accounting for regional variation in the appropriate terms. To address the two other parameters of *quality* and *recentness*, we further refined our search by targeting articles published in journals listed by the Chartered Association of Business Schools (CABS), Australian Business Dean Council (ABDC), the UK’s Association of Business Schools (ABS), and the Social Science Citation Index (SSCI). We extended our search to a variety of sources including ProQuest, ESBCO, books, webpages, and relevant journal articles. The original search was conducted in 2018 and updated in 2022.

## Theoretical framing of the influences shaping HRM practices

This section considers the three multilevel influences (macro-, meso-, and microlevel) that shape HRM practices in Asian contexts. These levels interact with each other and represent manifestations of practices or actions driven by the values and behaviors key decision-makers consider to be important for the operation of their business. Before delving into those, however, we state herewith the commonly understood goals of HRM, and investigate the extent to which these are found in an Asian context. Boxall and Purcell (2011) offer an excellent discussion focusing on the key goals of HRM. They assert that HRM must endeavor to balance economic goals (such as cost-effectiveness, flexibility, and creating a sustainable HR advantage) with socio-political goals (such as achieving social legitimacy, societal embeddedness, and the managerial prerogative) to deliver sustained value and remain relevant. Boxall (2012) further highlighted, based on studies of high-performance work systems, how a company should focus on balancing the economic goals of efficiency and performance with supporting its employees’ sustainable wellbeing. We propose that this is a useful starting point when analyzing what HRM typically sets out to achieve in an Asian context, and we overlay this with research on the National Business Systems (NBS) as a key influencer of not just HRM practices but also business practices (Edwards et al., 2022; Whitley, 1999; Witt & Redding, 2014).

In the seminal work in this field, Whitley (1999) noted a set of nine institutional dimensions in a country that affect the nature and extent of its business practices. Although largely for a European context, Whitley (1999) identified these as education and skills formation, employment relations, financial system, inter-firm networks, intra-firm dynamics, ownership and corporate governance, social capital (trust), and the role of the state. Witt and Redding’s work (2013, 2014) then mirrored Whitley’s original list of institutional dimensions. For the purposes of this analysis, we focus our attention on the following dimensions: Education and skills formation, employment relations, labor market dynamics at the intra- and inter-firm levels, and the role of the state, as these dimensions are central to HRM and key in any country. 

Keeping in mind the pervasive impacts of globalization, we also employ in this paper the convergence–divergence–crossvergence theoretical lens proposed by Ralston and colleagues (Froese et al., 2020; Mishra & Sohani, 2020; Ralston et al., 1997). This stream of research focuses on the extent to which, over time, through a confluence of convergent and divergent influences, firms experience varying degrees of crossvergence. More specifically, through globalization, convergence occurs when a “common or similar” set of political, economic, and managerial value systems (Brewster et al., 2015) has a standardizing impact on the management practices of a firm in a host country (Rowley & Benson, 2000). Divergence in management practices, on the other hand, occurs between the host and a parent firm when differences in their national regulations, government policies, national values, cultural beliefs, and other institutions are significant (Brewster et al., 2015). Finally, crossvergence, as defined by Ralston et al. (1997), is a process that arises “when an individual incorporates both national culture influences and economic ideology influences synergistically to form a unique value system that is different from the value set supported by either national culture or economic ideology” (p. 183). Through the above theoretical lenses, we set out to capture the national as well as international influences on HRM practices in Asian contexts. We begin by discussing the evolution and transformation of Indian HRM practices, followed by China, Vietnam, Kazakhstan, and the UAE.

## Historical evolution of HRM in Indian enterprises

The total workforce of India is estimated to be in excess of 400 million, and a vast majority of the workforce, nearly 90%, is in the informal sector (Datt & Sundram, 2014), where no formal HRM systems exist. The nature of HRM systems then is largely confined to the 10% of the workforce employed in the formal or organized sector. This includes small- and medium-sized enterprises (SMEs) as well as large corporations. The SME sector does not typically have a formal HRM infrastructure or policies. SMEs generally rely on hiring locals and employing largely informal, unstructured, and indigenous approaches to managing their staff, with major regional differences (e.g., Budhwar & Varma, 2011a; Malik et al., 2021a, 2021b; Saini & Budhwar, 2008).

As with any country’s evolution and transformation, India in its early stages of development actively pursued a state-led and regulated system of economic growth following the exit of the British in 1947, whose presence had adversely affected India’s social and economic prosperity. Like India, following the exit of the British, other countries in the Indian subcontinent such as Sri Lanka, Pakistan, and Bangladesh followed the path of deregulation and opening up their economies to foreign direct investment (Budhwar & Varma, 2011a; Chandrakumara & Budhwar, 2005; Khilji, 2004). Since instituting a major economic liberalization program in 1991, India has witnessed a major influx of human and monetary resources, mainly in the form of foreign investment in its economy. This has had major implications for HRM, especially as a number of multinational corporations have sought to exploit the existing pools of labor and also devise ways of retaining staff during the competitive and large-scale entry of multinational corporations (MNCs) (Budhwar & Varma, 2011b; Ramaswamy, 2003). Additional challenges on the supply side of labor were created when India’s newly minted engineering and medicine graduates were lured away from the Indian subcontinent to pursue further studies or work overseas (Baruch et al., 2007).

### Institutional dynamism and change

Another interesting change that occurred concerned the Indian educational system, which had established roots in the Anglo-Saxon educational system, given the presence of the British for many years. As India began to open up, many of its leading business and management schools adopted similar curricula to those of the USA and UK, leading to the proliferation of an Anglo-Saxon managerial mindset. While such an approach proved advantageous from a global human resource management (HRM) perspective, it also created challenges around integrating and embedding these practices and values with local employees (Budhwar, 2012). The extant literature on the formal and organized sector confirms that an established and formalized HRM function in Indian enterprises has existed for decades now (Saini & Budhwar, 2014), and the public sector has inherited many aspects of the British public service and legislative system. These early developments laid the foundations for a strong personnel and administrative function in India (Budhwar & Bhatnagar, 2009), along with the establishment of the Indian Institute of Personnel Management (IIPM) in Calcutta and the National Institute of Labour Management (NILM) and the Tata Institute of Social Sciences, both in Bombay (now Mumbai).

Early economic activity while still under British rule also saw a mirroring of UK common law, including the application of some employment legislation such as the Trade Unions Act in the early 1900s. Given the diversity of economic and political ideologies in India, and the scale of the workers to represent, several hundred local and regional trade unions were registered to represent the rights of their members. The country has over ten national unions, which typically have a highly adversarial stance in bargaining processes and are invariably linked to a major political party in the country (Saini & Budhwar, 2014), often also reflecting their ideologies. This active and relatively widespread proliferation of trade unions in India’s formal labor market has played a direct role in shaping the HR function. For example, with the emergence of a formal personnel function and the establishment of labor legislation, many commercial enterprises in the formal sector began to employ labor welfare officers as early as the 1920s. The remit of these officers was to deliver functional goals in the areas of labor welfare, industrial relations, and personnel administration.

The next stage of evolution of the Indian HRM system occurred in the mid-1970s, wherein the key focus of the personnel and administrative function shifted from welfare and administration to delivering the goal of a firm’s “efficiency,” and subsequently, in the 1980s, toward organizational development and human resource development (HRD). Following the economic liberalization programs of 1991, the Indian personnel management and industrial relations system witnessed a major set of reforms to allow businesses and industries flexibility in managing and organizing their labor policies. A case in point is the highly successful Indian software, information technology (IT), and business process outsourcing (BPO) industry, which employs a range of modern and globally comparable HRM practices (Budhwar et al., 2006a, 2006b, 2009). Yet, the changes instituted for this sector do not apply to foreign firms operating in other sectors in India (Björkman & Budhwar, 2007), leading to extensive variation in the nature and extent of HRM practices. This suggests the significant influence of a set of contingent factors at play (e.g., age, size, sector, ownership) and indeed the influence of India’s NBS on the HRM systems of Indian and foreign firms (Budhwar & Bhatnagar, 2009).

### Developments of modern HRM in the Indian subcontinent

It was not until the Indian economy’s high gross domestic product (GDP) growth rates of the late 1990s and mid-2000s that major transformative pressure was placed on the Indian HRM function to become more responsive, proactive, market-oriented, and innovative (see Budhwar & Bhatnagar, 2009). In fact, in some industries, such as the IT and BPO industry, the focus was more on developing multiple configurations of client-specific HRM within a single enterprise, with some common and harmonizing elements that cut across such a design (Malik & Rowley, 2015; Malik et al., 2017; Mathew et al., 2022; Pereira & Malik, 2015). The above transition suggests that from its early days to the present-day HRM, the Indian HRM system has evolved from administrative and welfare-oriented paternalism, through regulatory adherence, to adopting a strategic, proactive, and innovative human resource development (HRD) and management focus, aimed at delivering organizational performance through people (Budhwar & Varma, 2011b). This developmental focus has produced several iterations of a structured approach to competency development in India, each geared toward maintaining business dynamism and effectiveness (Saini & Budhwar, 2014). Yet, a major challenge faced by the HRM function in India is to change the mindset of the senior leadership, which is required if Indian enterprises are to best shape their HRM systems to help achieve their organizational objectives. Furthermore, in the Indian context, the presence of strong trade unions and industrial democracy in a number of industries, along with other forms of political activism, creates barriers to exercising managerial prerogatives concerning efficiency-focused transitions (Nankervis et al., 2013; Saini & Budhwar, 2014). Recently, with an increasing focus on digitization and the use of artificial intelligence (AI) for HRM, and India as a major global IT hub, there has been a proliferation of AI-assisted applications for HRM (Budhwar et al., 2022; Malik et al., 2021a, 2021b, 2022a, 2022b, 2022c) designed to improve a range of business- and employee-level outcomes, such as those concerning the employee experience and degree of engagement.

## Human resource management systems of Chinese enterprises

Elsewhere in Asia, the emerging economy and transition society of China provide a particularly interesting context in which to examine the divergence and convergence of HRM systems and practices. Traditional Chinese personnel management has been characterized by employment security (or the “iron rice bowl”) and egalitarianism (Ding et al., 2001). Yet, the ongoing market-oriented economic reforms have led to a greater performance orientation among Chinese firms.

We noted various perspectives in the literature and studies on China, but determined several mainstreams among these studies, such as a dual-concern model of HR systems evolving into performance- and maintenance-oriented HR subsystems, Confucian HRM, and a “hybrid system.” Gong et al. (2009) extended Katz’s and Kahn’s (1978) idea and developed a dual-concern model of HR systems, which evolve into two distinct subsystems: Performance- and maintenance-oriented HR subsystems. The former focuses primarily on developing HR and providing motivation and opportunities for the productive use of such resources. The maintenance-oriented HR subsystem, meanwhile, mainly concerns employee protection and equality. The authors suggested that maintenance-oriented HR subsystems are positively related to continuance commitment, while performance-oriented HR subsystems are positively related to affective commitment, which, in turn, enhances firms’ performance.

In another article, Warner (2010) coined the term “Confucian HRM” to explain how traditional Chinese values continue to influence the extent to which HRM has been adopted. He proposed a hybrid model that simultaneously includes HRM from Western societies and traditional Chinese values. Under this, social order is proposed to positively relate to harmony at work, and hierarchy is hypothesized to benefit the vertical linkages between supervisors and subordinates (Warner, 2010). Elsewhere in the literature, Su and Wright (2012) proposed that an effective Chinese HRM system is a “hybrid system” consisting of both commitment and control HR practices, which has much more significant positive effects on firms’ performance than American-style high-commitment and high-involvement working practices. The authors posited that an HR system focusing on basic control practices, as well as including commitment practices, yields better business results for Chinese enterprises than a Western-style high-performance working system. Interestingly, Liu et al. (2017) examined the effects of different types of HR systems by testing the moderating effect of ownership on the relationship between HRM systems and employee creativity. They found that employee-experienced performance-oriented HR systems were more positively related to employee domain-relevant skills when employees experienced stronger maintenance-oriented HR systems. In addition, employee-experienced maintenance-oriented HR systems more strongly augmented the positive relationship between employee-experienced performance-oriented HR systems and domain-relevant skills in privately owned enterprises (POEs) than in state-owned enterprises (SOEs). A key finding was that employee domain-relevant skills mediated the three-way interactive effect of employee-experienced performance-oriented HR systems, maintenance-oriented HR systems, and firm ownership (POEs vs. SOEs) on employee creativity. Employee creativity had a stronger positive relationship with firm innovation in POEs than in SOEs.

### Social and cultural factors affecting the human resource management systems of Chinese enterprises

Currently, studies commonly suggest that the main social and cultural factors influencing the HRM systems of Chinese enterprises can be classified into institutional factors, such as national and provincial policies, and national cultural factors, such as those under Confucianism and feudalism. Zhang (2012) suggested that HRM in China is influenced by two major factors: Critical *changes to policy-making* and *cultural* contributions, and also called for a cultural basis characterized by risk-taking and innovation. Yang (2012), meanwhile, discussed three major competing and merging ideologies: Confucianism, socialism, and capitalism, which have shaped Chinese culture. The author suggested that contemporary organizational behavior and management practices in China tend to reflect ideologies of the three cultural forces. To provide examples, Confucian values such as *guan-xi* and *de* (morality) can be seen to be related to managerial philosophies and practices in China. *Guan-xi* is said to influence organizational knowledge-sharing, which may affect employees' values and behavior. *De*, meanwhile, is commonly regarded as a key performance criterion, and morality and personal responsibility are also critical to social harmony and the equality system (Yang, 2012). Su and Wright (2012) pointed out that China has had an authoritarian culture for thousands of years in its long feudal history, which profoundly influences the thinking and behavior of Chinese people. In hierarchical and authoritarian cultures, empowerment HR practices are generally not so readily accepted either by managers or their subordinates. Looking ahead, Zhu et al., (2007a, 2007b) proposed that the national culture (based on Confucianism and feudalism) will have a unique and long-lasting influence on the emergence of HRM in China, which adds support to the attention we draw to a need to adopt convergence, divergence, and contextualization perspectives in related research.

### The nature and archetype of Chinese HRM practices

Using a dual-concern model of HR subsystems, Gong et al. (2009) identified commonly examined HR practices that apply to the middle-manager level and classified them into a two-factor structure of HR practices in Chinese firms, comprising maintenance- and performance-oriented HR subsystems. This model was supported by a factor analysis and comparison of measurement models. When examining middle managers' affective and continuance commitment to a firm as mediators, they found that a performance-oriented HR subsystem was positively related to a firm’s performance, and the relationship was mediated by affective commitment. In contrast, a maintenance-oriented HR subsystem had no significant relationship with a firm’s performance. It had a significant and positive relationship with the continuance commitment but no such relationship with the affective commitment.

Elsewhere in the literature, by contextually adapting the conceptual components of commitment-, collaboration-, control-, and contract-based HRM systems, Zhou et al. (2012) showed pluralistic structures of human resource management systems in China, consisting of two discriminant dimensions within each HRM system, and a high-order model encompassing all HRM systems. Based on this, they developed a new measure comprising four HRM systems each following specific practices. Some practices were adapted from multiple previous studies but synthesized with indigenous practices in the Chinese workplace, that is, they integrated Chinese context-specific HRM practices with the established concepts of HRM systems in the literature. For example, the HRM practices of value-based selection, intrinsic incentives, work-life benefits, and relationship maintenance were integrated with the high-commitment working system in China as these practices embodied the high-commitment characteristics of the Chinese workplace.

Rather than using the proportion of employees covered by certain HRM practices as the anchor in their survey, Zhou et al. (2012) asked respondents to rate the extent to which those practices had been adopted by the firm. The results showed that none of the four HRM systems was a single dimensional construct. Instead, each contained two discriminant dimensions. Specifically, commitment-based HRM contained affiliation-dependency and merit-enhancement dimensions; control-based HRM contained job-based-regulation and transactional-employment dimensions; collaboration-based HRM contained network-building and network-exploitation dimensions; and contract-based HRM contained contract-legitimacy and investment dimensions. This provided an in-depth exploration and clarification of the infrastructure of differential HRM constructs (Becker & Huselid, 2006). The results demonstrated a synchronous coexistence of multiple HRM systems within the Chinese workplace, which corresponded with the observation of hybrid HRM with Chinese characteristics (Cooke et al., 2021; Warner, 2008, 2010). Furthermore, the findings suggested that in Chinese firms, commitment- and collaboration-based HRM systems were positively related to the firm’s innovation. These two HRM systems, along with control-based HRM, were positively related to a firm’s cost constraints and bottom-line performance. The contract-based HRM system, however, did not significantly affect the firm’s performance, only partially reducing its costs.

The authors provided explanations for their findings from a dual-force perspective. On the one hand, the development of HRM in Chinese firms has undergone a process of so-called “modernization,” mimicking the HRM practices in North America, Japan, and Europe (Warner, 2008). On the other hand, practices that originated in traditional institutions and customary culture continue to play an important role (Tsui et al., 2004). These dual forces, plus the growing human capital heterogeneity in the Chinese labor market, cause so-called HRM pluralism or hybridism, in which multiple HRM patterns coexist and overlap within organizations. Using the typology approach, Su et al. (2018) developed a two-dimension model of employee governance in Chinese organizations. The two dimensions were named “eliciting employees’ commitment to the organization” and “achieving employees’ compliance with rules,” and they comprised four approaches to employee governance: Hybrid, bonded, disciplined, and unstructured. The results showed that a hybrid approach (high commitment and high compliance) produced the highest organizational performance compared to alternative governance approaches, suggesting that both commitment-eliciting practices and compliance-achieving practices are important for organizational performance in Chinese organizations.

## Macro-level business and institutional climate in Vietnam

As an emerging economy, Vietnam has experienced significant change at both the macroeconomic and organizational levels since its economic reforms, called *Doi Moi*, which started in the mid-1980s, aimed at transforming its communist, planned economy into a socialist, market economy. Attracted to Vietnam by its favorable investment laws, foreign MNEs established foreign-owned enterprises (FOEs) and joint ventures (JVs). The rapidly increasing number of such foreign-invested companies has put greater pressure on indigenous private companies and state-owned enterprises (SOEs), which must attend to their efficiency if they are to remain competitive (Zhu & Verstraeten, 2013).

Deeper involvement in free trade agreements such as the ASEAN (Association of South East Asia Nations) Free Trade Area (AFTA), US–Vietnam Bilateral Trade Agreement, and WTO membership has necessitated rapid and comprehensive changes at both the macro- and microlevels to enhance their competitiveness. In this context, the skilled, flexible, and well-educated workforce and its management, which are considered the most important sources of competitive advantage at both economic and organizational levels (Dmitrovic & Zupan, 2001; Porter, 1990), could not stand outside the development spiral. While labor costs are still low in Vietnam, developing a high quality of human resource and its management are seen as the keys to the country’s growth and development (Vo & Rowley, 2010).

Integration of foreign-invested enterprises into the Vietnamese economy since the 1990s has led a growing number of indigenous private enterprises and SOEs to recognize the importance of HRM. In accordance, they have started to adopt HRM “best practices” to improve their efficiency, flexibility, and competitiveness (Zhu et al., 2008). However, the context with respect to the relationship with the government, institutional constraints, industry, social environment, and organizational characteristics such as the ownership, company history, size, organizational culture, and strategy have significantly impacted which best practices have been applied and how (Vo & Rowley, 2010).

Previous studies found that ownership, size, sector, and market orientation are the key factors influencing the extent to which HRM practices are applied (e.g., Kamoche, 2001; Zhu, 2002; Zhu & Verstraeten, 2013). In one finding, better technology and higher international standards of HRM practices have been shown to represent advantages of JVs and MNCs when in competition with local companies (Froese et al., 2010; Kim et al., 2012). In response, under pressure to match that competitive advantage, a variety of local companies, especially more dynamic indigenous private ones, have adopted a range of HRM practices of their own. Zhu (2002) reported that high-tech, large, and export-oriented companies have adopted most HRM dimensions. In a further finding, SOEs that are transformed into joint-stock companies have been shown to be more likely to implement HRM practices than conservative SOEs set against the formalization of “new” HR practices. Furthermore, research has demonstrated that the adoption of HRM practices is not always a one-way process, but instead may be reciprocal. Reciprocity has been noted between foreign and local companies in terms of their HRM practices, with local companies adopting international standards in HRM practices, and MNCs and JVs following local rules and norms. To provide an example, Bartram et al. (2009) found that international JVs practiced cost-focused international HRM practices, as well as considered local customs around wages, welfare, and promotions.

Nevertheless, local Vietnamese companies, especially SOEs, generally adopt the widely used Western and Japanese HR practices to a lesser extent when compared to foreign-invested companies. Local companies’ lack of openness to adopting imported HR practices is proposed to derive somewhat from the perceptions of their owners. The misinterpretation of HRM as a synonym for “personnel management,” with connotations to the old form of personnel management practice, is still common among SOEs (Truong et al., 2010; Zhu et al., 2007a, 2007b). Often, local Vietnamese companies combine administrative tasks with personnel management, creating a so-called Division of Administration and Personnel Management, or they lack an HRM function entirely. If there are so-called personnel managers at local companies, they generally perceive administrative, operational (e.g., wages, social welfare calculations, and so on), and even secretarial works to be their main duties, in line with traditional personnel administration. They are rarely involved in or given responsibilities concerning HR-related key decision-making, such as that on planning, recruitment, promotions, bonuses, training and development, or employee retention (Truong et al., 2010). Further factors that leave the adoption of HRM by local companies lagging behind are the lack of a long-term view or professional competencies among the “short-term-oriented” management, which hampers the pursuit of strategic HRM. The management normally considers HRM initiatives as short-lived or quick-fix solutions rather than anticipatory tools designed to support future business development (Nguyen & Bryant, 2004).

Another major barrier to changing the people management system in Vietnam is that although the government has relinquished its control over recruitment and employment, with the aim of creating a more flexible people-management system as part of the reform agenda, there has been a relatively slow pace of transformation of “job for life” employment into a new, predominantly fixed-term contract employment system. Resistance to the transfer away from lifetime employment may stand as a residual effect of the old paternalist ideology, which remains rooted in local Vietnamese companies’ management. However, the introduction of a new fixed-term contract employment system, which derives from Western ideology, has gradually begun to change that perspective. In addition, the government has issued a new policy introducing a social insurance system, to replace the old “from cradle to grave” welfare system.

In addition, a fundamental factor profoundly influencing various aspects of and the degree to which HRM practices are adopted in emerging economies such as Vietnam is how traditional cultural and value systems combine with political and legal environments such as the socialist market economy. Although economic reform is assumed to reduce Party influence on enterprises, political networks form a readily accessible structure for informal bargaining, which may still proceed through personal connections, generating problems ranging from unpredictability to corruption (Zhu & Fahey, 2000). The findings of Zhu and Verstraeten (2013) demonstrated that the recruitment and selection processes of local Vietnamese companies remain highly relationship-based, with most of their respondents mentioning the possibility of bypassing the processes. Furthermore, Vietnamese cultural traditions, as well as upholding the importance of personal connections, place great emphasis on organizational and personal commitment, along with harmonious working environments, which can prevent the full deployment of functional flexibility initiatives and reductions to the number of staff (Zhu, 2005). Furthermore, Confucianism can be said to still have a certain impact on Vietnamese organizations’ HR philosophy, for instance, contributing toward their collective-oriented incentives and organizational hierarchies. The philosophy of collectivism is also demonstrated by their group-oriented approach to work, with an emphasis on teamwork. Group-based decision-making, quality control, and incentives are common managerial practices in Vietnam.

However, one area in which local Vietnamese firms perhaps have the edge concerns their marked shift from traditional seniority-oriented pay systems, with an emphasis on “age and experience,” toward a competency-based pay system (Zhu & Verstraeten, 2013). Local private companies have become the leading organizations to widely implement a bonus system based on performance evaluation results, whereas FOEs and JVs are still novices in the domain of applying a competency-based pay system to replace a seniority-oriented one.

In Vietnam, in general, different contexts with respect to the relationship with the government, institutional constraints, industry, social environment, and organizational characteristics such as ownership, company history, size, organizational culture, and strategy have significantly impacted which imported best practices are applied and how. Our literature review highlighted that the most dynamic indigenous companies are private firms that have rapidly changed their HRM practices; the performance of these firms exceeds that of SOEs and traditional private firms, as a result, and some have already caught up with FOEs and JVs (Zhu & Verstraeten, 2013). Although FOEs and JVs still lead in terms of the adoption of HRM practices, they face tough challenges from local Vietnamese companies that are becoming increasingly competitive. Recently, research on technological adoption in Vietnamese workplaces has increased, including the use of AI-assisted tools to enhance knowledge-sharing and job performance (Nguyen & Malik, 2021a, 2021b, 2021c; Nguyen et al., 2021a, 2021b).

## Human resource management systems in Central Asia—the case of Kazakhstan

Central Asia comprises the countries of Kazakhstan, Kyrgyzstan, Tajikistan, Turkmenistan, and Uzbekistan. These five countries, among the so-called “Stans,” were formerly part of the Soviet Union. Despite their common history, the countries are diverse in terms of ethnic and cultural backgrounds, the pace of economic development, and their share of natural resources, among other factors. Central Asia’s economic activity is centered on heavy and light industry, paired with mining in Kazakhstan and irrigated agriculture in the south of the region. This central part of Asia has long possessed large volumes of oil and natural gas: Kazakhstan is the region’s leading oil producer and Turkmenistan is its main gas exporter.

In terms of economic development, Central Asia's economic growth amounted to 2.7 percent in 2018 and 4.9 percent in 2019 (World Bank, 2022), mainly due to rising oil prices. In 2018, the World Bank reported that the economies in Central Asia were continuing to improve their business climate, seeking to create jobs and spur on further growth. Membership of the WTO (Kyrgyzstan since 1998, Tajikistan since 2013, and Kazakhstan since 2015) has helped certain Central Asian states to modernize customs administration and technical standards, and reduce non-tariff barriers to trade and investment barriers” (Batsaikhan & Dabrowski, 2017). However, their economic improvements have been impeded by widespread corruption, weak formal institutions, and slow development of democracy.

Around 15–20% of the government expenditure of the countries in Central Asia goes on education (on par with the OECD average). Yet, the quality of human capital, which is fundamental to how these countries will advance in the new economic environment, remains low. Despite the high number of university graduates, there is a continual lack of qualified applicants for work positions (those with a diploma from a recognized university, knowledge of foreign languages, and additional functional training or work experience). An individual possessing the desired knowledge and skills may receive two or three job offers at once, which contributes to a high level of job-hopping, especially in regions with high FDI. A further challenge is that the new realities of business environments (digital revolution, disruptive trends, volatility of markets, etc.) require 21st-century skills and significantly higher levels of human capital than in the past—something the Central Asian education system cannot really offer. Most of its higher education institutions have been privatized and are now largely concerned with profit generation, while considering faculty development a low priority.

### Influences on HRM

In Central Asia, HRM—as business professionals in Western societies understand it—has only recently started to take hold in the management discourse and in practice. As in all other Asian countries, MNCs have certainly played a significant role in reshaping the HRM practice in the Central Asian region, especially in terms of the emergence of mimetic pressures to adopt “best practices” and new approaches (Morley et al., 2016). Researchers studying this development often fail to see past “Western” theories of HRM, questioning only the extent to which these theories can be applied in emerging-market contexts. Their findings seldom provide evidence of a unique approach to HRM emerging in such a context. Instead, what is commonly inferred is a difficult balancing act between standardization and customization (Edwards et al., 2016), leading in many cases to dynamic, uncertain, and unplanned forms of hybridization of HRM policies and practices (Minbaeva et al., 2007).

The Soviet past still significant influences working practices in Central Asia. As in Russia, the degree of local adaptation of HRM depends on the extent of an organization’s embeddedness in the pre-transformation socialist system of employment relations (determining the segment of post-socialist capitalism they now fall under).^1^ For instance, de novo enterprises will more widely adopt the global “best practice” approach to HRM, while state-owned companies will operate with locally adapted, indigenous HRM practices (Andreeva et al., 2014). Russia still plays a significant role in defining the HRM landscape in the Central Asian region. In fact, many similarities exist between HRM in Central Asia and Russia. This could be due to their common past and the origins of their HRM in the Soviet model of employment relations—*otdel kadrov.* The latter included the artificial maintenance of full employment (“labor hoarding,” Filatotchev et al., 1996), low wage differentiation, the predominance of a wage scale system, a mandatory performance evaluation called *attestatsiya* (attestation), and numerous nonmonetary benefits (e.g., medical services, education, cultural events, food, vacations at company resorts, and awards for special achievements). As Fey et al. (1999) stated, during Soviet times, employees were viewed as a cost rather than a resource, and so organizations did not invest in employee training or skill development. This mindset is still present in the majority of organizations operating in the Central Asian region (Morley et al., 2016). Notably, just like in Soviet times, HR departments mainly deal with personnel administration and record-keeping, and are regarded as a control function with low involvement in strategy.

As in other Asian countries, evidence of multivergence (Malik et al., 2017) can be found in certain sectors. For example, the HRM practices of oil and gas companies in Kazakhstan are similar, regardless of the companies’ origins. Furthermore, it is common for all Central Asian countries to face challenges with HRM in the public sector. In terms of regional influences, Russia has had the greatest influence on the development of HRM. Interestingly, another powerful neighbor in the region—China—was not as welcomed at the beginning of the HRM transition, but attitudes toward Chinese management have evolved over time. Minbaeva and Muratbekova-Touron (2011) studied the experience of two consecutive acquisitions of an oil and gas company in Kazakhstan: First by a Canadian, relatively unknown firm, and second, by a state-owned Chinese giant. At the time of the first acquisition, the underdeveloped state of the local HRM, coupled with a knowledge and skills vacuum, provided a “fat land” for North American HRM. At the time of the second acquisition, the emergence of Kazakhstani HRM (a hybrid of old-style Soviet and Western-based (North American and European) approaches) had been reported (see Minbaeva et al., 2007). The degree of social integration (or rather the absence thereof) created a poor perception of the attractiveness of the acquirer, along with the economic development of the acquirer’s country of origin compared to that of the previous owner. The employees of the acquired company did not trust or respect the new owners and lacked confidence in them (“we *cannot learn much from the Chinese*”) compared to the previous owners. Yet, the situation and attitudes changed over time. Now the total trade between China and Central Asian countries has surpassed Central Asia’s trade with Russia, with commodities dominating. Chinese firms learned from their early experiences in Central Asia and gradually adjusted their approaches to managing human resources in Central Asian subsidiaries.

There are no notable emerging-market MNEs originated from the region. Yet, every Central Asian country has a big and powerful, majority state-owned company, with which foreign MNEs interested in commodities enter into joint ventures or establish close collaboration. In such joint or collaborative companies, HRM is heavy on administration and exercising control, taking a top-down approach. Training and development are formalities, and in many instances, the companies take a reactive approach to training, so their training strategies respond to the particular needs at a time. Recruitment and selection in such companies are influenced by kinship and nepotism. Despite continued efforts to modernize state-owned firms, they remain plagued by widespread nepotism, that is, the practice of recruiting and promoting based on family ties and connections rather than competence. There is a saying: “*There are no love affairs in state-owned companies because all employees are relatives.*”

Today, nepotism affects most HRM-related decision-making. Hotho et al. (2013), from an institutional logic perspective, studied the effects of clannism on recruitment and selection practices in Kazakhstan. They proposed that we must greatly consider *when* and *how* human resource professionals experience community obligations in work situations. Drawing on the literature on social cues, they asserted that interactions with community members in the workplace can serve as social cues that expose human resource professionals to communal obligations, even in workplaces where meritocratic HRM practices are the norm. They also observed decoupling between the strategies employed by human resource professionals to cope with nepotistic community obligations, and the actual recruitment intentions of these professionals.

In Kazakhstan, *rushyldyq* can help to secure access to various essential goods: Economic, social, or political. Minbaeva and Muratbekova-Touron (2013) gave examples of kin connections’ uses when seeking to find a job or forge business connections. In Uzbekistan and Tajikistan, the question “Which region are you from?” is a common way to start a conversation with a potential business partner. Tunçer-Kılavuz (2009) interviewed politicians from those two nations and found that the “region is taken into consideration in the allocation of posts in politics and that there are informal quotas for people from different regions” (pp. 326–327). As such, kinship—or more generally, a person’s informal network—sometimes offers professional advantages.

## HRM practices in the United Arab Emirates

The seven Emirates, Abu Dhabi, Dubai, Sharjah, Ajman, Um Al Quwain, Ras Al Khaimah, and Fujairah, came together in 1971/1972 to form what is the modern-day United Arab Emirates. According to the International Monetary Fund (IMF), the UAE is classified as a high-income developing economy, one of the most developed and economically important countries in the Middle East. The GDP per capita in the UAE was last estimated at 38,661 USD in 2020 (IMF, 2022).

The government’s and leadership’s primary objective for the UAE is to build and develop an innovative and modern knowledge-based economy. An amalgam of resources and competitive advantages offered by each of the Emirates has helped the UAE to pursue this strategy. For example, Abu Dhabi’s considerable oil and gas resources, alongside a deliberate effort to develop and expand the public infrastructure and services throughout the country, have helped stimulate growth in the region (Suliman, 2006). Dubai, on the other hand, has traditionally built itself up as a trading hub by investing heavily in world-class ports and airports. More recently, it leveraged these assets to emerge as a booming tourist destination, and it now seeks recognition as a center for financial services and technology. It has further strategically positioned itself as a pioneer of e-government services in the Middle East (e.g., Zhao et al., 2012). Additionally, Dubai has benefitted from creating free zones where 100 percent foreign ownership is allowed, which has propelled exports and helped the UAE to become a thriving business hub in the Middle East. The next largest Emirate is Sharjah, which has traditionally focused on its cultural and educational initiatives, providing a more traditional counterpoint to Dubai’s constant modernization. Sharjah is also home to many expatriates, who live there because of its more traditional cultural and educational initiatives and lower costs of living, including rentals. Emirati culture can be described as one based on a traditional tribal way of life, strongly influenced by Islam (Zhao et al., 2012). It shares many aspects of a common Gulf culture emphasizing the importance of religion, family, education, and success (Abdalla & Al-Homoud, 1995; Al Bahar et al., 1996; Klein et al., 2009). However, the modern-day UAE is particularly seen as an important economic and cultural hub, and as one of the most liberal countries in the region, with high tolerance and acceptance of other religions and faiths.

Article 20 of the UAE Constitution specifically commits the national government to “endeavour to ensure that employment is available for citizens and to train them so that they are prepared for it.” Furthermore, UAE Vision 2021 lists “skilled human capital” as the first of seven strategic enablers, consistent with the role of HR, which is described as “building strategic capabilities” to achieve strategic goals (Scott-Jackson et al., 2011; Wright & McMahan, 1992). However, academic research on HRM in the Arab world, in general, is lacking (Budhwar et al., 2018). This can mainly be attributed to difficulty in finding reliable data and the lack of a research culture in the region (Forstenlechner & Rutledge, 2010; Harry, 2007).

The most unique and unusual characteristics of the UAE labor market are its extensive reliance on expatriates and its dual nature. As the UAE began its modernization and strategic economic development, the existing skills, learning, training, development, and education levels of locals could not meet the rapidly increasing needs of a fast-changing and expanding country, which led to high demand for skilled and knowledgeable foreign labor (Fasano-Filho & Goyal, 2004). Traditionally, at the upper end of the labor market, Western expatriates from the UK, the US, Continental Europe, and Australia were hired. This primarily served to bring in management skills and technical expertise, which these expatriates possessed through their experiences in their home countries, and which they could pass on to their Arab colleagues. Meanwhile, at the other end of the spectrum, there was a large demand for workers from low-wage countries to fill positions within the construction and services sectors, in unskilled and semi-skilled occupations. These workers mainly originated from the Indian subcontinent, that is, from India and Pakistan.

Workers from the Indian subcontinent including Bangladeshi, Nepalese, and Sri Lankan migrants, alongside those from India and Pakistan, who came to collectively make up 60% of the lower end of the labor market. Interestingly, workers from other Arab countries only came to represent 13% of the UAE workforce, with local Emirati nationals even fewer. Expatriate workers at the lower end of the market were, and still are, contracted for a fixed period and sponsored for a work visa by their employer, with limited mobility. By 2010, native citizens made up only 12% of the total population and 4% of the UAE workforce (Forstenlechner & Rutledge, 2011). As such, the composition of the UAE labor market is unique and complex, which makes it worthy of research. A recent study by Collings et al. (2018) investigated host-country nationals’ (HCNs’) characteristics and willingness to help self-initiated expatriates (SIEs) in the UAE. Findings from their study show that UAE HCNs have a tendency to categorize SIEs based on ethnocentrism and collectivism, though they provide information and social support to the SIEs.

Local Emirati nationals prefer jobs that offer high wage levels, short working hours, and good benefits, such as government jobs. In desirable, employee-friendly job positions, workers enjoy certain facilities and privileges, such as decent remuneration and benefits packages and guaranteed job security, even in the private sector. Given the lack of these for others, local Emirati workers and expatriate workers are not comparable. However, it is not necessarily better to be among the former, for the phenomenal growth in the number of expatriates has not created sufficient opportunities for locals, meaning they face heightened unemployment. As a result, Emiratis are under increasing pressure to train professionally and gain relevant skills, knowledge, and qualifications. An estimated 200,000 young Emirati locals will have entered the labor market over the past ten years armed with such qualifications, just what the country needed (Toledo, 2013). Today, the UAE is pursuing an Emiratization policy, seeking to minimize its reliance on foreign nationals and restore an Emirati workforce. This provides organizations and managers a unique opportunity to contribute to the local agenda by designing, implementing, and monitoring efficient local talent management processes (Randeree, 2009). According to the Federal Authority for Government Human Resources (2010), Emiratization rates in the federal government and ministries reached 57 and 67 percent, respectively, at the end of 2010. Similarly, Emiratization of nationalized banks was 34%, and of non-national financial institutions was 21% (Duncan, 2014), rates that we can only assume have increased since.

The UAE has the highest number of published research articles on HRM among the Arab countries, which signals that HRM is attracting growing and evolving attention from both researchers and business leaders. The growth of both local and multinational corporations in the UAE has crucially underpinned the country’s modern and innovative HRM practices. Furthermore, a growing number of foreign and local universities enroll students on campuses in the UAE, which led to an increase in experienced HR professionals in the country. However, so far, the HRM function in the UAE has not demonstrated its value, nor effectively contributed to the achievement of organizations’ strategic goals. Many generally accepted HR practices have yet to be implemented fully, and there are significant variations in the ways in which HRM strategies and practices are formulated and implemented in the UAE.

Our critical review of the literature leads us to conclude that the HRM function faces four key challenges in the UAE, which are common to other Arab countries (Budhwar et al., 2018). The first challenge is to effectively align HR strategies and practices with organizations’ strategic goals. Then, the second challenge is to improve the effectiveness of HR processes, particularly in key areas identified as most important by businesses, since HRM departments in the UAE are currently struggling to establish objective and efficient HR systems (Al Ariss, 2014; Suliman, 2006; Yaseen & Khanfar, 2009; Yaseen, 2013). The third challenge is to improve the professionalism of HR practitioners, since today, HR professionals in the UAE lack relevant experience and education. To provide an example, in research by Scott-Jackson et al. (2014), on average, respondents reported four years of experience working in HR roles, but much of this experience was administrative and sometimes barely related to HRM. A fourth significant challenge is related to the development of HRM processes that are relevant to the UAE and meet the specific needs of national or organizational cultures and management models in the country. In summary, the HR environment in the UAE is complex due to the evolving education system, the existence of a dual labor market, the extensive reliance on an expatriate workforce, the diverse and multicultural labor pool, the challenges of engaging and retaining employees, the increasing pressure to pursue Emiratization, and evolving UAE government regulations.

## Discussion

Having separately analyzed HRM in five countries, we now provide an integrated understanding of overall HRM in Asia. To begin with, our literature review suggests significant diversity and also certain overlaps in terms of the goals of HRM functions. These goals share similarities with Boxall’s and Purcell’s (2011) classification of social, political, and economic goals of HRM. Table 1 below outlines commonalities and differences in the emphases and contents of Asian HRM goals. Our findings suggest there is hybridity in terms of the diverse and sometimes competing goals held in Vietnam, Kazakhstan, and the UAE. While Asian countries often share similar social, economic, and political goals of HRM, the exceptions are Vietnam and Kazakhstan. Furthermore, the workforce composition in the UAE is unique given that most of the country’s residents and workers are expatriates.

**Table 1:** Goals of HRM across countries

Countries	Goals of HRM			
	Social	Economic	Political/power	Our comments
China	Employment security	Market-oriented efficiency	Egalitarianism	*Dual system representing maintenance- and performance-oriented systems*
India	Welfare and regulatory	Efficiency, competitiveness and innovation	Paternalistic	*Multiple configurations of social, economic and community goals*
Vietnam	Supporting welfare and life time employment mindset	Efficiency, flexibility and competitiveness	Paternalistic	*Caught between push for economic growth and modernization amidst strong institutional inertia in traditional sectors*
Kazakhstan	Kinship and communal	Cost effectiveness focus	Engineered social goals, e.g., Labour hoarding	*Old versus sector represents the traditional Soviet model of a bleak house of HRM while the new economy sectors embrace global best practice goals of HRM*
UAE	Localization (emiratization) but over dependence on expatriates	Dual labor market	Federal government driven	*An impetus on economic growth and modernization, but needs to streamline challenges of localization and strategic HRM best fit and best practices*

Our review also revealed significant diversity in terms of the multiple levels of influence that shape the nature and extent of HRM practices. At the macrolevel, we classified the influences into indigenous or modern, which we found a useful distinction to make as the early stages of economic growth and evolution of HRM in these countries were reflective of the dominant indigenous philosophical and ideological approaches. Similar macrolevel indigenous influences were noted in China, Vietnam, and the UAE, whereas India and Kazakhstan had somewhat different macrolevel factors shaping their HRM practices (see Table 2 above). At the mesolevel, the influences were classified into two groups: Contingency variables, such as the industry, ownership, size, age, and maturity of the organization, and the external influence of the parent MNC or the JV partner’s global best practices in HRM. Finally, there were also some similarities in the microlevel factors shaping HRM practices. Employees’ relational capital and networks in the workplace context were particularly noted to help shape certain HRM practices. In turn, their individual capabilities and agency were influenced by macrolevel values, for example, in India, the excessive focus on having a strong service mindset. That is an artifact of the previously held Gandhian values, which now serve to underpin the nation’s “customer-centricity.” Similarly, in the UAE, indigenous influences stem from the traditional tribal way of life, strongly influenced by Islam, though that is now evolving, with strong shifts in the most liberal countries in the region toward high tolerance and acceptance of other religions and faiths. It was interesting to also note the unique case of Kazakhstan, where the regional affiliation of an individual contributes—positively or negatively—to determining their employment outcomes.

**Table 2:** Levels of Influence on HRM practices

Countries	Levels of influence		
	Macro	Meso	Micro
China	***Indigenous influences:*** Confucianism and feudalism ***Modern Influences:*** Market-oriented economy with socialist characteristics (Cooke, 2011)	Contingency factors: ownership (e.g., State-owned enterprises), size and national importance Hybrid organizations MNC influences	*Guanxi* (Confucian value of individual's social network and relationships) influences employees' values and behavior
India	***Indigenous influences:*** Socialist and secular values and strong industrial democracy Political intervention Paternalistic ideology ***Modern Influences:*** Liberalization agenda and global integration	Contingency factors: Mainly in the form of industry- and sector-specific models of HRM Multivergence at an industry level MNC influences	Duality of individual *Jugaad* (finding a creative fix to a tricky problem) and Gandhian values of customer-centricity leads to an external locus of control in framing behaviours and actions
Vietnam	***Indigenous influences:*** Paternalistic ideology Aspects of Confucianism and collectivist philosophy ***Modern Influences:*** Doi moi Liberalization	Diverse set of contingency factors: ownership, size, industry, culture, strategy, history MNC influences	Relationship-based approaches Personal connections Organizational and personal commitment Experience and age Team-based approaches
Kazakhstan	***Indigenous influences:*** ‘Stans’ and Soviet model Weak formal institutions Nepotism and kinship ***Modern Influences:*** Partial adoption of global HR best practices	Communal obligations Hybrid forms of firms (Traditional SOEs and SOEs with minor adaptations) Multivergence at an industry level Partial MNC Influences	Assess via *Rushyldyq* Family ties Personal connection Geographical affiliation Relational capital
UAE	***Indigenous influences:*** Based on traditional tribal way of life and is strongly influenced by Islam ***Modern Influences:*** Knowledge driven economy and cultural hub One of the most liberal countries in the region with high levels of tolerance and acceptance of other religions and faiths	Localization obligations Hybrid forms of firms (Local firms, MNEs and SMEs) Corssvergence and Multivergence in knowledge-based industries Convergence and MNE Influences in traditional industries Divergence in SMEs	Emiratization Wasta Emphasizing the importance of religion, family, education and success on one hand, but then also evidence of high levels of tolerance and acceptance of other religions and faiths

At the next level of analysis, we delved into how four key aspects of a country’s dominant NBS (Whitley, 1999; Witt & Redding, 2014) have shaped the nature and extent of its HRM practices (see Table 3 below). Each of the four dimensions affected how employers and key stakeholders determined the rules of employment. For example, the role of the state varied from developmental to predatory and regulative. Employers’ (or the dominant coalition of decision-makers’) leeway and freedom to make choices at the enterprise level can be curtailed or expanded depending on the role of the state (Kochan et al., 1994). Similarly, differences in the development of a country’s education system affect how its citizens can effectively participate in the labor market. India, for example, is known for its prowess in technical education and for having a generally well-educated and English-speaking workforce. Thanks to these factors, it has sustained strong performance in a number of knowledge-intensive service-oriented business sectors over the past few decades. Kazakhstani and Vietnamese economies, meanwhile, are somewhat constrained from entering and fully participating in knowledge-intensive industries, since their workforces lack the education required for the two countries to take advantage of global opportunities. Then, we have the UAE, which is quickly moving from a resource-dependent economy toward a knowledge-based economy, but has to cope with HRM challenges in this transition. The aspects covered in the summary above have implications for the design and implementation of HRM practices in Asia, which must be attuned to the setting if the country is to attract and retain talent. If that can be achieved, then investing in human resource development practices will help the case study countries to overcome their deficiencies in the supply side economics of human capital.

**Table 3:** Influence of NBS Elements on HRM practices

Countries	NBS influences			
	Role played by state	Educational system	Industrial relations system	Social capital
China	Economic and market regulator role declining role as a key employer developmental state (Carney & Witt, 2014) MNCs have leeway	Strong educational system reliant on a hybrid model	Largely unilateral one union (‘ACFTU) (Cooke, 2011) lack of independence of the union state regulated	Formalized, rule based and interpersonal relations
India	Interventionist developmental state (Carney & Witt, 2014)	Very strong and diverse Strong supply side though emerging issues in quality and lacking in sustained R&D investments	Adversarial stance by unions highly regulated bilateral and trilateral	Formalized, rule- based and interpersonal relationships
Vietnam	Developmental and predatory state (Carney & Witt, 2014; Witt, 2018)	Weak skills formation Medium level of educational outcomes (Witt, 2018)	High union membership, communist party affiliation short-tenure contracts (Witt, 2018)	Interpersonal relationships (Witt, 2018)
Kazakhstan	Predatory state	Evolving supply side human capital formation but a weak demand side	Artificial full employment, labour hoarding	Strong interpersonal relationships and geographical affiliation
UAE	Transitioning from a predatory state to interventionist developmental state (Carney & Witt, 2014)	Expatriate driven, with the major population being expats	Illegal to have unions	Formalized, rule based and interpersonal relations

Meanwhile, the extent of unionization and its nature will directly determine the rules of employment in each country. Today, China has a single trade union that is regulated and managed by the state, while in India, a democratic country, unions have different political and ideological affiliations, different densities and membership sizes by industry, and are generally perceived as independent of the state. As such, in a bargaining setting, India’s trade unions deliver different outcomes for their members compared to those in China. In India, one can expect varied employment rule-making for different industry sectors, and strong adversarial stances during the bargaining process, whereas China, in contrast, is characterized by homogeneous employment laws and regulations and compliance with the rule of the state.

A final factor to note is that the social sphere, which held relevance in all Asian countries studied, but played diverse roles. Kazakhstan, for instance, has inherited socialist Soviet approaches of artificially re-engineering or “beefing up” employment numbers to achieve social legitimacy. Aside from this, social capital and relational business practices were noted to be employed across the board, though the actual practices reported and the extent of their effect on HR varied.

The purpose of our study was to contribute to context-specific scholarship and research. We assert that thorough, descriptive understandings of each Asian region will provide the basis for developing a thematic and relational understanding of each country in terms of how their multiple levels of influences shape and affect indigenous HRM practices to converge, diverge, or form some novel amalgam of HRM practices. Table 4 portrays divergence and convergence (or crossvergence and multivergence) across different countries through the lens of the convergence–divergence–crossvergence framework (Al Ariss & Sidani, 2016; Froese et al., 2020; Mishra & Sohani, 2020; Ralston et al., 1997).

**Table 4:** Examining the convergence-divergences-crossvergence (CDI) thesis

Countries	CDC thesis		
	Convergence	Divergence	Crossvergence/ Multivergence
China	Weak levels of convergence	Strong divergence	Inter-regional crossvergence
India	Weak levels of convergence	Inter-regional divergence	Industry-specific Multivergence
Vietnam	Medium levels of convergence	Strong as highly heterogeneous HRM practices but weak for start-ups and private sector firms	Weak levels of crossvergence Medium level for new industry and private sector firms
Kazakhstan	Medium levels of convergence	Strong divergence due to significant indigenous influences	Weak levels of crossvergence
UAE	Convergence and MNE influences in traditional industries	Divergence due to localization obligations (Public sector, local firms and SMEs)	Corssvergence and Multivergence in knowledge-based industries

Our analysis suggests weak convergence of HRM practices for the two fastest-growing global economies of China and India. Conversely, for Vietnam and Kazakhstan, we observed medium overall convergence, while in the UAE, convergence was paired with MNE influences on traditional industries. When it came to divergence of HRM practices, China seemed to experience a strong level of divergence, whereas, not surprisingly, with greater diversity, inter-regional divergence was found. Likewise, there was evidence of strong divergence in Vietnam due to the highly heterogeneous nature of HRM practices in the country, though only weak divergence was seen for startups and private-sector firms. In comparison, for Kazakhstan, we found evidence of strong levels of divergence, which could have arisen due to the significant local indigenous influences. Interestingly, for the UAE, there was strong evidence of divergence in the public sector and among local firms and SMEs, which could be attributed to localization effort, that is, Emiratization. Lastly, when it came to crossvergence/multivergence, China showed evidence of inter-regional crossvergence, which could be strongly attributed to the levels of influence of MNEs, paired with local indigenous practices that are still strong and divergent in certain parts of China. In comparison, for India, there was evidence of certain industry-specific multivergence, especially in the IT/BPO industry (Malik et al., 2017, 2020). The evidence for crossvergence in Vietnam, meanwhile, was weak; however, this was seen somewhat for new industries and private-sector firms. In the UAE, levels of crossvergence and multivergence were evident in new knowledge-based industries, which are growing rapidly as the UAE is transitioning into a knowledge-based economy.

In sum, based on the critical review and analysis presented above, we can conclude that the main influences on HRM practices in Asia are the regionalization of economies, national business systems, industry, global MNEs, and individual-level predispositions, which collectively shaped and variously influenced the nature and extent of HRM practices in the Asian countries studied. We found a mix of convergence, divergence, and crossvergence/multivergence as each country and region in Asia offered a distinctive amalgam of national business systems, and the varied ways in which HRM practices responded to multilevel influences were complex and evolving. This work contributes to the literature by presenting a comparative and relational understanding of HRM practices in five key Asian regions. In the future, more such studies should be conducted to further unravel and separate out this complex, evolving, and dynamic research area, that is, HRM in Asia.

## Footnotes

1 Martin (2008) identified various segments of post-socialist capitalism: State-owned enterprises, privatized companies, de novo enterprises (established during the transformation period), and international companies (e.g., subsidiaries of foreign MNEs).
